# Novel Colon Targeted Drug Delivery System Using Natural Polymers

**DOI:** 10.4103/0250-474X.40346

**Published:** 2008

**Authors:** V. Ravi, T. M. Pramod Kumar, Siddaramaiah

**Affiliations:** Department of Pharmaceutics, J. S. S College of Pharmacy, S. S Nagar, Mysore - 570 015, India; 1 Department of Polymer Science and Technology, S. J. College of Engineering, Mysore - 570 006, India

**Keywords:** Colon targeting, natural polymers, pectin, inulin and shellac

## Abstract

A novel colon targeted tablet formulation was developed using pectin as carrier and diltiazem HCl and indomethacin as model drugs. The tablets were coated with inulin followed by shellac and were evaluated for average weight, hardness and coat thickness. *In vitro* release studies for prepared tablets were carried out for 2 h in pH 1.2 HCl buffer, 3 h in pH 7.4 phosphate buffer and 6 h in simulated colonic fluid. The drug release from the coated systems was monitored using UV/Vis spectroscopy. *In vitro* studies revealed that the tablets coated with inulin and shellac have limited the drug release in stomach and small intestinal environment and released maximum amount of drug in the colonic environment. The study revealed that polysaccharides as carriers and inulin and shellac as a coating material can be used effectively for colon targeting of both water soluble and insoluble drugs.

In recent times, colon targeted drug delivery systems have gained importance for the systemic delivery of protein and peptide drugs. This is because the peptide and protein drugs get destroyed or inactivated in acidic environment of the stomach or by pancreatic enzymes in the small intestine^1^. Drug targeting to colon is also useful when a delay in drug absorption is desired from therapeutic point of view, such as treatment of diseases that have peak symptoms in the early morning like nocturnal asthma, angina or arthritis^2^,^3^. Among the different approaches to achieve colon specific drug delivery, the use of polymers, specifically biodegraded by colonic bacterial enzymes holds promise^4^,^5^.

The important bacteria present in the colon such as *Bacteroides, Bifidobacterium, Eubacterium, Peptococcus, Lactobacillus, Clostridium* secrete a wide range of reductive and hydrolytic enzymes such as β-glucuronidase, β-xylosidase, β-galactosidase, α-arabinosidase, nitroreductase, azoreductase, deaminase and urea hydroxylase. These enzymes are responsible for degradation of di-, tri- and polysaccharides^6^,^7^. Pectin is a polysaccharide obtained from plant cell walls. Inulin is a polysaccharide, which is obtained from plants such as onion, garlic, chicory and artichoke. Being soluble in water, pectin is not able to shield its drug load effectively during its passage through the stomach and small intestine. Hence a coat of a considerable thickness is required to protect the drug core in simulated *in vivo* conditions. The aim of the present study was to develop pectin based matrix tablet with sufficient mechanical strength and promising *in vitro* mouth-to-colon release profile. The matrix tablets were coated with inulin and shellac to target the tablets to colon. The coated tablets were evaluated for weight gain, coat thickness and *in vitro* dissolution studies.

The drugs selected for the study were indomethacin and diltiazem hydrochloride that are used in treatment of early morning arthritis and angina respectively. Indomethacin was obtained as a gift sample from Bhavani Pharmaceuticals, Kanpur and diltiazem from Divis Laboratories, Hyderabad. Pectin, inulin and shellac were procured from Loba Chemie, Mumbai. Pectin with 65% esterification was used for the present study.

Pectin matrix tablets of diltiazem hydrochloride were prepared by wet granulation process. Accurately weighed quantities of drug, polymer (pectin) and binder (PNP K-30, 3% w/w) were mixed in a mortar. Required quantity of the solvent (ethanol) was added and the same was mixed thoroughly to form a mass suitable for granules. The dough mass was passed through sieve # 22 to form granules which were dried in an oven at 50°. The granules were mixed with required quantities of diluent (lactose) and lubricant (talc, 3% w/w) and were compressed to form tablets in a 10 station rotary tablet machine (Rimek, Mumbai) using 9 mm round concave punches at an optimum pressure. Three formulations of 500 tablets each were prepared with different polymer (pectin) concentrations viz., 25, 50 and 75% w/w (DF1, DF2 and DF3) of the tablet. The total weight of each tablet was 310 mg and contains 60 mg of diltiazem hydrochloride. The same parameters were taken for the preparation of indomethacin tablets (IF1, IF2 and IF3).

The prepared pectin matrix tablets were coated with inulin by solution coating. Inulin coating solution (10% w/v) was prepared in hot water using glycerol (3% w/w of inulin) as plasticizer. Coating of the tablets was done in a conventional coating pan (Ram Scientific Suppliers, Bangalore) at an inlet temperature of 55°, pan rotation speed of 15 rpm, spray pressure of 4 kg/cm^2^ and a spray rate of 10 ml/min. A pilot type spray gun (Bullows 630) fitted with a 1 mm atomizing nozzle was used to spray the solution. The influence of degree of coating of inulin on pectin-drug blend system have been studied i.e, 2, 3 and 4% w/w of inulin coat on matrix tablets. After drying, the inulin coated tablets were further coated with shellac to a weight gain of 2.5% w/w. Shellac coating solution (10% w/v) was prepared in ethanol using glycerol (3% w/w of shellac) as plasticizer. *In vitro* release studies were carried out for 2 h in 1.2 pH HCl buffer, 3 h in 7.4 pH phosphate buffer and 100 ml of simulated colonic fluid (SCF) in a 200 ml beaker for another 6 h^8^,^9^. 4% w/v of rat cecal contents in pH 7 saline phosphate buffer, incubated for 24 h was used as SCF^10^,^11^. The drug release was monitored using UV spectrophotometer (Shimadzu 1601, Japan) by measuring the absorbance at wavelengths of 237 nm and 320 nm for diltiazem and indomethacin respectively.

The principal IR absorption peaks of diltiazem at 2966, 2837, 2393, 1679, 839 and 781 cm^−1^ were obtained in the spectra of the pure drug as well as pectin-diltiazem complex indicating that no chemical interaction occurred between the diltiazem and the polymers used. The principal IR absorption peaks of indomethacin at 3400, 3000, 1695, 1450, 1230, 950 and 835 cm^−1^ were obtained in the spectra of the pure drug as well as pectin-indomethacin complex indicating that no chemical interaction occurred between indomethacin and the polymers used. The coated tablets were found to have a thickness of 5±1.2 mm and a hardness of 5.5±0.5 kg/cm^2^. The assay tests conducted showed that all the formulations were having the drug content of 98±1.2%. *In vitro* studies carried out revealed that the formulations containing 25 and 50% of pectin could not show sustained release whereas the formulations DF3 and IF3 (75% pectin) showed sustained drug release over a period of time. Formulations containing 25 and 50% w/w of polymer released entire drug within 6 and 8 hrs of dissolution respectively. On the other hand tablets containing 75% w/w of polymer showed only about 90% of drug release at the end of 11 h of dissolution (Fig. 1). That means the order of drug delivery with reference to polymer concentration is 25 > 50 > 75 % w/w.

**Fig. 1:**
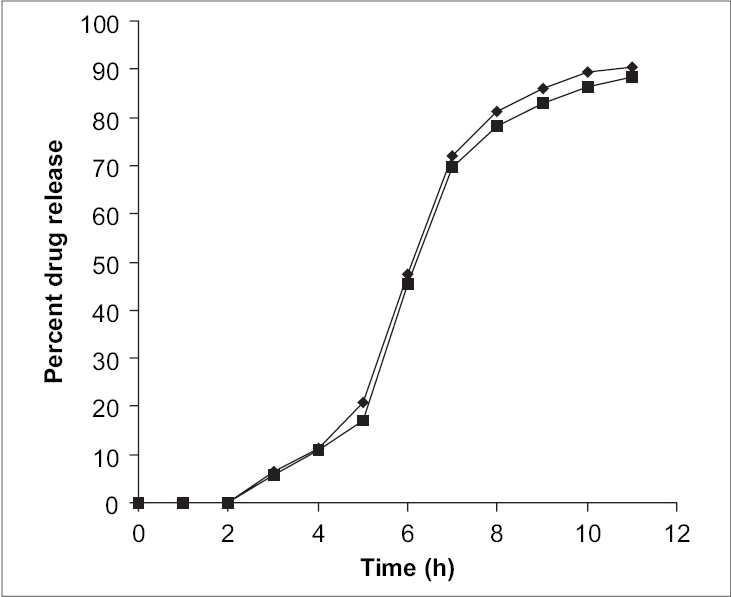
Drug release profiles of DF3 and IF3 formulations. Release profile of pectin-diltiazem HCl tablet (DF3) and pectinindomethacin tablet (IF3) for 11 h.

All the formulations were found to show no drug release in pH 1.2 buffer (stomach environment) and showed a little release of about 18±1.5% of the drug in pH 7.4 phosphate buffer and released the remaining amount of drug in the colonic environment. Release studies revealed that shellac coat (2% w/w) prevented the drug release in stomach environment and out of the inulin coated formulations, the formulations coated with 4% w/w inulin showed less drug release in small intestinal environment. On reaching the colonic environment, the inulin coat gets degraded and the drug gets released. SEM photograph of the coated tablets is shown in Fig. 2. The tablets were found to have an inner coat (inulin) of 30 μm and an outer coat (shellac) of 20 μm.

**Fig. 2:**
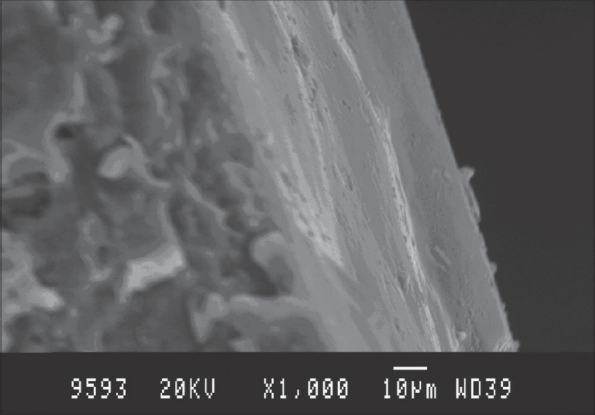
SEM photograph Pectin-drug matrix tablet. Cross section of tablet coated with inulin (4% weight gain) and shellac (2% weight gain) as revealed under scanning electron microscopy.

From this study it can be concluded that the prepared novel tablet formulation can be used for colon specific drug delivery for targeting both water soluble and water insoluble drugs. Inulin and shellac together in form of film coat has proved to be effective for colon targeting of tablets.
